# The Sense of Being Watched Is Modulated by Arousal and Duration of the Perceptual Episode

**DOI:** 10.1177/2041669517742179

**Published:** 2017-12-04

**Authors:** Vera M. Hesslinger, Claus-Christian Carbon, Heiko Hecht

**Affiliations:** Abteilung Allgemeine Experimentelle Psychologie, University of Mainz, Germany; Department of General Psychology and Methodology, University of Bamberg, Germany; Research group EPÆG (Ergonomics, Psychological Æsthetics, Gestalt), Bamberg, Germany; Department of General Psychology and Methodology, University of Bamberg, Germany; Research group EPÆG (Ergonomics, Psychological Æsthetics, Gestalt), Bamberg, Germany; Abteilung Allgemeine Experimentelle Psychologie, University of Mainz, Germany

**Keywords:** arousal, eyespots, microgenesis, observation cues, sense of being watched

## Abstract

The mere presence of a depiction of eyes can elicit a sense of being watched in the perceiver. To this date, the factors affecting the intensity of this sense of being watched, however, have not been investigated. In the present experiment, we tested the impact of two potentially relevant variables: arousal (manipulated using specific musical pieces) and duration of the perceptual episode (manipulated using presentation times of 200 ms and 10 s, respectively). We asked participants to report how intensely they felt being watched while we exposed them to various observation cues ranging from human eyes to surveillance cameras. We found that, on average, reported intensities were higher if participants were in a state of relatively higher arousal and if the perceptual episode during which the respective observation cues were presented lasted long enough (10 s) to allow more than a first glance. Scientific and practical implications are briefly discussed.

Warning signs that exhibit eyes or cameras are frequently used by owners of private homes as well as in public spaces to discourage acts of vandalism and other crimes (see examples depicted in Figure 1). It has indeed been shown that the mere presence of a depiction of human eyes can elicit a sense of being seen or watched (Pfattheicher & Keller, 2015) and reduce negative behaviors such as littering (e.g., Ernest-Jones, Nettle, & Bateson, 2011) and theft (Nettle, Nott, & Bateson, 2012).

**Figure 1.**
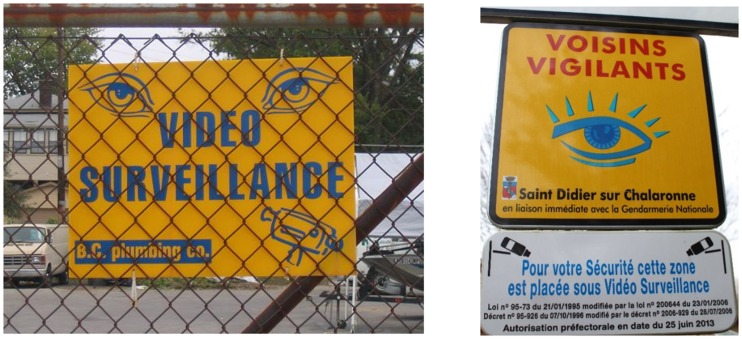
Examples for the use of observation cues in private and public spaces. Images under creative commons license (CC BY-SA 3.0/4.0), retrieved from https://commons.wikimedia.org; left: video surveillance sign by Quadell; right: panneau “voisins” vigilants" à Saint-Didier-sur-Chalaronne by Chabe01; both originals have been cropped.

Basically, the sense of being seen or watched can be understood as having the impression that someone else is around, potentially seeing, observing, and maybe even reacting to one’s own actions. It is thought to originate from processes relevant in the context of human social interaction, namely the processes responsible for detecting conspecifics and (implicitly) decoding information about other minds (see, e.g., two-process model of humans making use of eye-cues proposed by Grossmann, 2017). The sense of being watched triggered by these processes presumably is an essential prerequisite for the specific behavioral responses and changes mentioned earlier. Most likely, other observational cues work in a similar way as eye-cues.

A pioneering experiment investigating the impact of eye-cues on behavior was reported by Haley and Fessler (2005). They found that the mere presence of a depiction of eyes sufficed to significantly increase participants’ generosity in a dictator game. In subsequent experiments, in the laboratory as well as in the field, a comparable positive effect of eye-cues on socially relevant behaviors could be replicated (e.g., Bateson, Nettle, & Roberts, 2006; Burnham & Hare, 2007; Keller & Pfattheicher, 2011; Oda, Niwa, Honma, & Hiraishi, 2011; Panagopoulos, 2014; Rigdon, Ishii, Watabe, & Kitayama, 2009). The reliability of the eye-cues effect, however, is still under debate, as a narrow set of conditions might be required (e.g., sudden or brief exposure, meta-analysis by Sparks & Barclay, 2013; null effects found in other cases, e.g., Carbon & Hesslinger, 2011; Fehr & Schneider, 2010; Northover, Pedersen, Cohen, & Andrews, 2017; Raihani & Bshary, 2012; Tane & Takezawa, 2011). To reconcile the heterogeneous findings and to determine favorable conditions, we need to extend our knowledge regarding contextual features and characteristics of the cues and the perceivers, which are crucial for a reliable occurrence of the effect. This involves identifying and specifying factors that primarily affect the intensity of the sense of being watched elicited by eyes and other observation cues. With the present work, we experimentally focus on two potentially relevant variables: Arousal and duration of the perceptual episode during which a perceiver is confronted with eyes and other observation cues.

## Arousal

According to the response-facilitation model as described by Allen, Kenrick, Linder, and McCall (1989), a higher general arousal facilitates the dominant response that is normally elicited by a given stimulus. This facilitating effect is thought to be independent of whether the true source of the arousal is salient to the acting subject. Accordingly, we suggest that the sense of being watched induced by images with surveillance cues, such as human eyes, will be more intense under conditions of relatively higher arousal. Exploiting known effects of music on arousal (e.g., Dillman Carpentier & Potter, 2007; Thompson, Schellenberg, & Husain, 2001), in the present experiment we induced arousal with music.

## Duration

Perception is a dynamic process. This implies that the percept is not necessarily stable over time. According to the microgenetic approach (e.g., Bachmann, 2000), even on a time scale as short as about 100 ms, the percept evolves and runs through a series of different instable states. After the initial phase of the first glance, the percept potentially evolves and changes further. Caputo (2010) showed, for instance, that the percept of one’s own face seen in a mirror under dim light conditions can change dramatically over the course of 10 min, with illusory impressions usually starting to occur after less than 1 min. More than half of Caputo’s participants reported to have seen substantial deformations of their own face, and about one third reported seeing an unknown person. With regard to these and related findings that point to the dynamic nature of perception, we suggest that the perception of observation cues, and the sense of being watched which they elicit, is prone to change over time as well. In the present experiment, we compared a very short *first glance* perceptual episode of 200 ms (which should prevent the execution of saccades—with exception of express saccades; cf. Fischer & Ramsperger, 1984) with a perceptual episode of 10 s (which is long enough for closer inspection and first elaborative steps). We thus aimed to get a first idea of the temporal development of the sense of being watched within a relatively restricted time scale. We did explicitly not use perceptual episodes beyond this time scale, as Sparks and Barclay (2013) reported that exposures of several minutes or longer might corrupt the eye-cues effect.

## Method

### Participants

Forty-two students (3 males; age *M* = 20.6 years, range = 18–31 years) participated in the experiment for partial course credit. They all had normal or corrected-to-normal visual acuity and normal color vision, as tested by short versions of the Snellen eye chart test and the Ishihara color vision test, respectively. They were treated in accordance with the Declaration of Helsinki and gave written informed consent before the experiment.

### Design and Material

**Figure 2.**
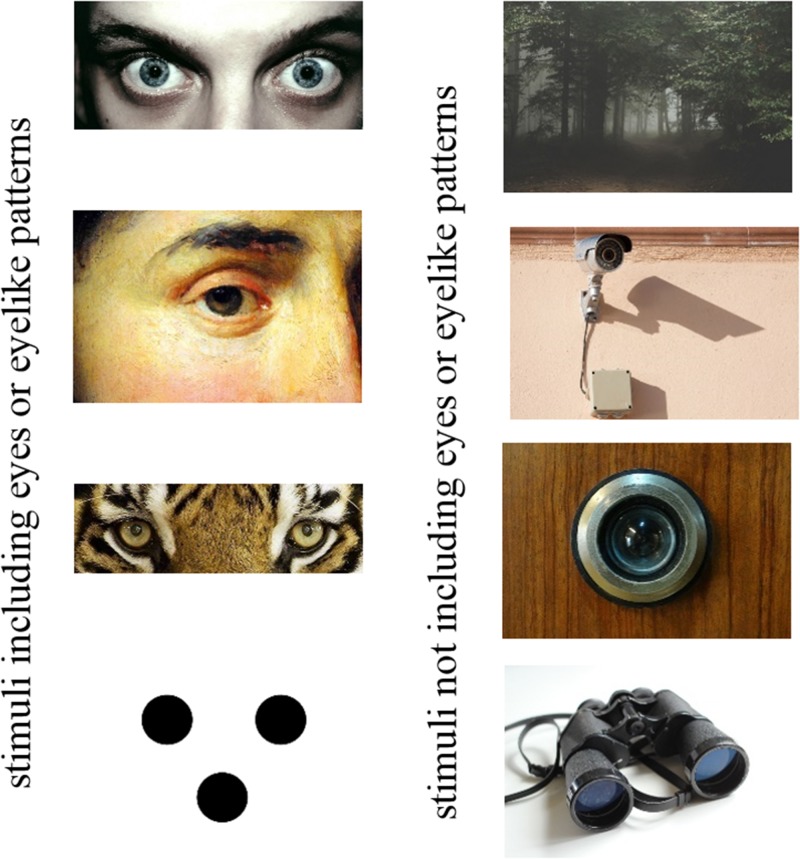
Exemplary observation cues used as stimuli in the present experiment. Images under creative commons license (Creative Commons CCO), retrieved from https://pixabay.com, except for the stylized face pattern (left column, bottom) which we generated based on a stimulus used by Rigdon et al. (2009).

We used a fully crossed 2 × 2 repeated measures design to test the impact of the factors *music-induced arousal* (lower and higher) and *duration of the perceptual episode* (Presentation Time [PT] = 200 ms and PT = 10 s) on the intensity of the participants’ sense of being watched, as elicited by observation cues. As stimuli, we used depictions of 42 different objects and scenes showing, reminding of, or being related to seeing and observation, for instance, a pair of watching eyes, a surveillance camera, or the uncanny view into a dark forest where one might feel watched (see Figure 2 for some exemplary stimuli).^1^ The stimuli were standardized with regard to their width (800 pixels at 72 dpi). They were presented using a Dell Desktop PC with an Eizo Color Graphic CG245W TFT monitor (wide screen; 24.1” diagonal, resolution = 1,920 × 1,200 pixels; color depth = 32 bits per pixel; and refresh rate = 60 Hz) using the latest available version (1.10.1630) of the experimental software *Experiment Builder* by SR Research. To manipulate the participants’ arousal level, we used the song *Weightless Part 1* (on the album *Weightless/Ambient Transmissions Vol. 2* released in 2012, Just Music, London), which was specifically created by the British band Marconi Union to relax the listener (cf. media coverage, e.g., Passman, 2016), and the composition *A Night on the Bald Mountain* by Modest Mussorgsky (played by the Los Angeles Philharmonic under conductor Esa-Pekka Salonen, released in 2006, Deutsche Grammophon, Berlin), which had successfully produced higher levels of arousal before (see Balch & Lewis, 1996). The pieces of music were played in an endless loop each using a portable MP3-player with stereo circumaural headphones. To assess personality variables that are related to reactions to or preferences for different arousal levels, we used a German translation of the Brief Sensation Seeking Scale (BSSS; Hoyle, Stephenson, Palmgreen, Lorch, & Donohew, 2002), a short German version of the Big Five Inventory (BFI-K; Rammstedt & John, 2005), and the German version of the Anxious Thoughts Inventory (AnTI; Hoyer & Rebstock, 1996). The items of the questionnaires were presented on the test computer using the open source online survey tool LimeSurvey.

### Procedure

Each participant completed three main phases: two test phases (T1 and T2) separated by a break of 5 min and a posttest phase. The two test phases basically had the same structure, the only difference being the piece of music that was played: Half of the participants heard the lower arousal piece in T1 and the higher arousal piece in T2; for the other half of the participants, the sequence was reversed. At the beginning of T1 and T2, respectively, participants were seated in front of the test computer and received verbal instructions about the rating task that followed. The experimenter also explained that they would hear some music via headphones and that the room would be darkened during the rating to prevent external acoustic and visual distraction. The true function of the music was not mentioned in the instructions. If the participants did not have any further questions, the experimenter asked them to put on the headphones, started the music and the experimental program, turned off the lights, and left the room. Except for the monitor of the test PC, there was no artificial source of light in the room; natural light from the outside was prevented from entering by a black blind covering the only window of the room. On the screen, a brief statement announced that the rating task would start in a few minutes. Six minutes later, the task started while the music kept on playing. The task comprised 42 trials, during each of which one stimulus was presented. The order of presentation was randomized. Each trial showed the following time course: Presentation of stimulus for 200 ms, immediate indication of intensity of one’s sense of being watched on a 7-point scale (1 = *I do not at all feel like I am being watched*, 7 = *I feel very much like I am being watched*), and presentation of the same stimulus for 10 s, immediate indication of intensity of one’s sense of being watched. To indicate the intensity of their sense of being watched, participants were asked to use the respective keys on the keyboard. As soon as the participants had seen and rated all stimuli, each test phase ended automatically. During the break between the test phases, the experimenter came back into the room and turned on the lights to test the participants’ handedness using a German translation of the Edinburgh Handedness Inventory (Oldfield, 1971) as well as their visual abilities using a Snellen eye chart and a short version of the Ishihara color vision test. In the posttest phase, participants evaluated the valence of all stimuli in randomized order on a scale ranging from 1 (*negative*) to 7 (*positive*) and answered the items of the personality questionnaires. Both tasks were realized on the same test computer used for T1 and T2. After the experiment, participants were verbally debriefed by the examiner.

## Results

**Figure 3.**
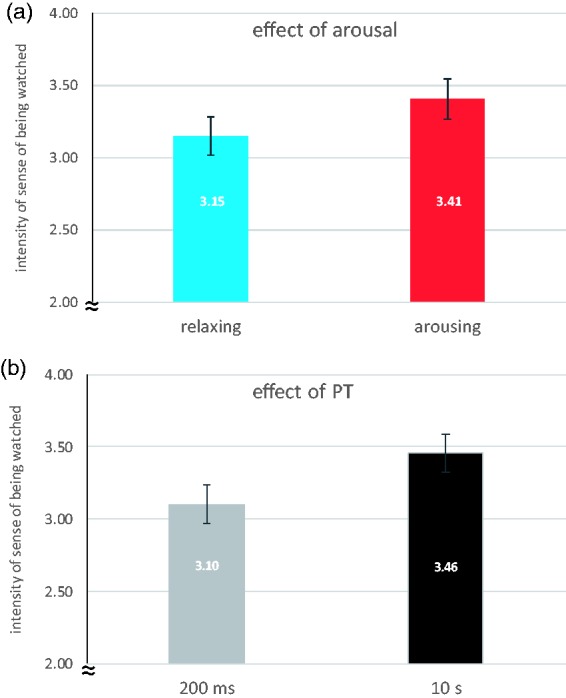
Mean intensity of the participants’ sense of being watched in the relaxing (lower arousal) vs arousing (higher arousal) music conditions (a) and in the short versus long duration conditions (b) measured using a 7-point scale ranging from 1 = *I do not at all feel like I am being watched* to 7 = *I feel very much like I am being watched*. Error bars indicate ±1 standard error of the mean calculated as adjusted values according to Morey (2008).

Using a 2 × 2 repeated measures analysis of variance, we tested the impact of the experimentally manipulated factors *music-induced arousal* (lower and higher) and *duration of the perceptual episode* (PT = 200 ms and PT = 10 s) on the intensity of the participants’ sense of being watched. We found significant main effects of music-induced arousal, *F*(1, 41) = 8.81, *p* = .005, $\eta_{\text{p}}^{2}$^ ^= .177, and duration of the perceptual episode, *F*(1, 41) = 21.32, *p* < .001, $\eta_{\text{p}}^{2}$ = .342. There was no significant interaction of the two factors, *F*(1, 41) < 1, *p* = .416, *ns*. The intensity of the participants’ sense of being watched induced by the stimuli was significantly higher when arousal was relatively higher, and when the stimuli had been shown for 10 s instead of 200 ms (see Figure 3).^2^

Additional item-based analyses of the correlations between the intensity of the participants’ sense of being watched and the perceived valence of the stimuli that participants had rated post hoc were run for all four combinations of arousal and duration of the perceptual episode. The results showed a significant negative relation between intensity of the participants’ sense of being watched and stimulus valence (−0.51 ≤ *r*s ≤ −0.44 and .001 ≤ *p*s ≤ .003): A more intense sense of being watched was associated with a less positive subjective valence. The causal direction can, of course, not be identified on basis of the present data and will thus not be further elaborated here.

To test for potential modulating effects of the assessed personality variables, we ran separate regression analyses for the brief and long duration of the perceptual episode. Personality variables (neuroticism as measured by the BFI-K, sensation seeking as measured by the BSSS, concerns about wrong behavior in presence of others as measured by the AnTi) were taken as predictors. The delta between the intensity of the participants’ sense of being watched under relatively higher versus lower arousal was taken as dependent measure. The analyses revealed that neither of the personality variables significantly explained variance in the dependent measure (*p*s ≥ .165).

## Discussion

Depictions of eyes and other observation cues can elicit a sense of being watched. With the present experiment, we gained some first insights about factors that affect the intensity of this sense of being watched. We found that the intensity was higher when participants were in a state of relatively higher music-induced arousal, as compared to a very reduced sense of being watched in a state of lower arousal. This is in line with the response-facilitation model which predicts effects of arousal on judgment and behavior (see Allen et al., 1989). Further, perceptual episodes of longer duration also yielded a more intense sense of being watched. This effect was even more pronounced than that elicited by the experimental manipulation of arousal in the present experiment. We suggest that the brief first glance our participants received of the observation cues (lasting merely 200 ms) sufficed to elicit a mild sense of being watched, maybe in terms of an automatic response. The longer second glance (lasting 10 s), however, provided enough time to inspect and elaborate the individual observation cues along several potential dimensions: While inspecting the stimulus for 10 s, observers might have made a higher number of eye fixations, which allowed them to assess a greater number of details of each cue. The participants may further have used the extra time to observe or register their personal reaction to the cues. Thus, besides being more detailed, the resulting percepts were probably also tinted by (more complex) associations, reflections, and self-observations, which may have intensified the participants’ sense of being watched. It is also conceivable that the participants started into a symbolic social interaction with the virtual looker, or that the observation cue triggered the simulation of actual social interactions. Both processes would be plausible sources of a more intense sense of being watched.

As the current experiment constitutes a first attempt to investigate context factors that affect the intensity of the sense of being watched, as elicited by observation cues, our findings have some obvious limitations and raise several new issues for future research on the topic. One limitation lies in the gender bias in our sample which comprised mainly female participants. Although the eye-cues effect does not seem to be affected by the participants’ gender in general, we cannot rule out gender-dependent differences in the behavioral response to eye-cues (e.g., Rigdon et al., 2009). Future gender-balanced studies might be in a better position to assess whether and how the intensity of the sense of being watched elicited by eyes and other observation cues is affected by the participants’ gender.

Concerning the manipulation of the participants’ state of arousal, we have relied on earlier works that reported effects of music on participants’ current arousal level (e.g., Dillman Carpentier & Potter, 2007; Thompson et al., 2001). There are other methods that have effectively been used to manipulate arousal. Allen et al. (1989), for instance, used fear-inducing information or physical exercise. If the response-facilitation model is indeed adequate, such alternate arousal induction methods should provide comparable results. The use of only two musical pieces in the present experiment is compatible with our initial hypothesis; however, it prohibits the conclusion that it was indeed a general arousal that produced the effect. For instance, the limited variety of stimulus properties inevitably increases the probability that the results are confounded by the characteristics of the stimuli that are unrelated to arousal but nonetheless selectively triggered associations or aesthetic feelings that altered the sense of being watched.

Regarding the duration of the perceptual episode, we cannot tell for sure which of the processes suggested earlier brought about the more intense sense of being watched in the case of the relatively longer episodes. It is hard to ascertain whether participants used the extra time to more closely inspect and elaborate on the stimuli or whether they reflected on their own reactions and relations to these stimuli. Asking participants to provide descriptions of what they have seen and thought during the briefer and longer perceptual episodes, for instance, may be problematic as such retrospective descriptions can be biased by a posteriori rationalizations and false memories. In the present experiment, we used perceptual episodes of two different durations. Future experiments should further vary the duration of the perceptual episodes to determine in more detail how exactly the intensity of the sense of being watched evolves over time. Such a variation should explicitly also approach longer exposure times to assess the potential saturation or habituation point, at which the intensity of the sense of being watched may decrease again (cf. Sparks & Barclay, 2013).

For scientific and practical reasons, we should continue to investigate which factors affect the sense of being watched in the presence of different observation cues. Regarding the scientific reasons, knowing more about these factors will contribute to the scientific debate on the reliability of the eye-cue effects on socially relevant behavior. Specifically, such findings can help to understand why an eye-cues effect is not found in certain instances. In the present experiment, the participants’ sense of being watched reached a medium level of intensity. Under conditions that differ from the ones implemented here, the level of intensity might be lower. It is possible that there is a threshold of intensity below which the elicited sense of being watched is too weak to affect behavior. From a practical perspective, it is important to know which circumstances can intensify the sense of being watched, such that behavior modification will ensue. Extending this knowledge is an important step on the road toward effective means of reducing or preventing social misconduct, such as vandalism. Their efficacy is tightly bound to the potential and limits of the cues that can elicit a sense of being watched.

## Notes

1 Three stimuli each belonged to the following categories: human adult eyes (pair), child’s eyes, animal eyes, single human eye, frontal view of a human head and upper torso, patterns found in nature reminiscent of eyes, eye-like artificial patterns, door spy, binoculars, photo camera, observation camera/CCTV, drone, dark space where one might be watched, scene with lights indicative of being in the focus of observation.

2 For reasons of variability, we used 14 different categories of observation cues in the present experiment (see Method section). The experiment, however, was not specifically set up for testing differential effects of these different categories on the participants’ sense of being watched. Therefore, we refrained from further analyzing the data with regard to such effects using inference statistics. As the intensity of being watched elicited by different kinds of cues is nevertheless of great interest for understanding the eye-cues effect, we inspected the descriptive statistics to get some clue regarding this point. The results indicate that, on average, the intensity of the sense of being watched was highest for stimuli showing eyes of humans or animals (human adult eyes, child’s eyes, animal eyes, single human eye, and frontal view of a human head and upper torso; *M* = 4.30). The lowest intensity, in contrast, occurred for stimuli showing eye-like patterns found in nature or artifacts (*M* = 1.79). Stimuli from the remaining categories, including inter alia stimuli showing technical devices used for observation, elicited intensities that lay in-between (*M* = 3.11).
